# Sexual assault resistance education for university women: study protocol for a randomized controlled trial (SARE trial)

**DOI:** 10.1186/1472-6874-13-25

**Published:** 2013-05-23

**Authors:** Charlene Y Senn, Misha Eliasziw, Paula C Barata, Wilfreda E Thurston, Ian R Newby-Clark, H Lorraine Radtke, Karen L Hobden

**Affiliations:** 1 Department of Psychology/Women’s Studies Program, University of Windsor, 401 Sunset Avenue, Windsor, Ontario N9B 3P4, Canada; 2 Department of Public Health and Community Medicine, Tufts University, 136 Harrison Avenue, Boston, MA 02111, USA; 3 Department of Community Health Sciences, University of Calgary, 3280 Hospital Drive NW, Calgary, Alberta T2N 4Z6, Canada; 4 Department of Psychology, University of Guelph, Guelph, Ontario N1G 2W1, Canada; 5 Department of Ecosystem and Public Health, University of Calgary, 3280 Hospital Drive NW, Calgary, Alberta T2N 4Z6, Canada; 6 Department of Psychology, University of Calgary, 2500 University Drive NW, Calgary, Alberta T2N 1N4, Canada

**Keywords:** Sexual assault, Rape, Resistance, Education, Intervention

## Abstract

**Background:**

More than one in six women will be sexually assaulted in their lifetimes, most by men they know. The situation on university campuses is even more startling, with as many as 1 in 4 female students being victims of rape or attempted rape. The associated physical and mental health effects are extensive and the social and economic costs are staggering. The aim of this randomized controlled trial is to determine whether a novel, small-group sexual assault resistance education program can reduce the incidence of sexual assault among university-attending women, when compared to current university practice of providing informational brochures.

**Methods/Design:**

The trial will evaluate a theoretically and empirically sound four-unit, 12-hour education program that has been demonstrated in pilot studies to have short-term efficacy. Three of the four units provide information, skills, and practice aimed at decreasing the time needed for women to assess situations with elevated risk of acquaintance sexual assault as dangerous and to take action, reducing emotional obstacles to taking action, and increasing the use of the most effective methods of verbal and physical self-defense. The fourth unit focuses on facilitating a stronger positive sexuality from which women may resist sexual coercion by male intimates more successfully. The trial will extend the pilot evaluations by expanding the participant pool and examining the long term efficacy of the program. A total of 1716 first-year female students (age 17 to 24 years) from three Canadian universities will be enrolled. The primary outcome is completed sexual assault, measured by *The Sexual Experiences Survey - Short Form Victimization* instrument. Secondary outcomes include changes in knowledge, attitudes, and skills related to the process of sexual assault resistance. Outcomes will be measured at baseline, 1 week, 6, 12, 18, and 24 months.

**Discussion:**

The results of the trial will be used to produce a maximally effective sexual assault resistance education program that can be adopted by universities, to assess whether aspects of the program need to be strengthened, and also to indicate how long the effects of the program last and at which point in time refresher sessions may be necessary.

**Trial registration:**

ClinicalTrials.gov NCT01338428

## Background

### Epidemiology and impact

By conservative estimates, between 18 and 25 percent of women will experience rape or attempted rape in their lifetimes (i.e., experience sexual penetration by the use of threats, force or drugging) [1-3]. If sexual assault not including penetration (i.e., forced, threatened or drugged non-consensual sexual touching) and sexual coercion (i.e., using tactics other than physical force or threats, such as “continual arguments and pressure”) are included, the percentage increases dramatically [4-6]. The incidence of sexual assault and coercion on Canadian and American university campuses are even more startling [6-8]. As many as 1 in 4 female students are estimated to be victims of rape or attempted rape during the relatively short time they are enrolled in post-secondary education [8]. First year students are most at risk [9], probably because university entrance is a transition time for adult intellectual, social, and sexual development, both healthy and unhealthy.

The physical and mental health effects of rape and other forms of sexual coercion have both acute and chronic negative consequences for victims, as well as extensive social and economic consequences. Physical consequences include unwanted pregnancies and sexually transmitted diseases, including HIV and Hepatitis C, [10], cigarette smoking, and alcohol and drug consumption [11,12]. Short and long-term psychological consequences include depression, post-traumatic stress disorder, suicidal ideation, lack of sexual enjoyment, and fear [13-15]. In Canada alone, sexual assault and other forms of violence against women cost at least $1.5 billion each year in health-related expenses [16] and substantially more in social services, legal processes, and lost time from employment [17]. Fear of rape also affects the quality of life of a high proportion of women who are not rape victims [18-20]. This fear is connected to precautionary strategies that restrict women’s movements at certain times and in certain spaces [21-23], and consequently limits women’s employment and recreational opportunities [20,22].

Girls and women between the ages of 14 and 24, regardless of their sexual orientation, are most at risk of being sexually assaulted by a man [1,3,24-26]. It, therefore, makes sense that efforts to protect women from sexual assault should take place prior to, or within, this ten-year time window. Although many sexual assaults occur prior to age 18 [27,28], restrictions on the types of sexual content that can be presented in schools make them a difficult site for implementing and evaluating rape prevention programs. In contrast, university campuses are ideal environments as students are adults, and therefore the restrictions are fewer.

### Rape prevention efforts on university campuses

There has been a long history of educational efforts to attempt to prevent rape on and off campus [29,30]. Historically, the most common approach on university campuses, due in large part to structural practicalities, has been to present coeducational audiences with correct information about rape (e.g., most rapes are committed by male acquaintances, undermining rape myths such as that women ‘provoke’ rape if they wear revealing clothes, etc.). These “universal” approaches try to reach both men and women but are not tailored for either [31] and have been shown to result in temporary improvement in rape knowledge and attitudes at best [32-35]. Moreover, most of these programs do not measure incidence or severity of sexual assaults (perpetration or victimization) following the program. In Canada, at the time the present trial began, universities’ rape prevention education appeared to range from provision of brochures to a five minute section of a play done for student orientation (e.g., *Single & Sexy*, University of Windsor) to 60–90 minute workshops for students (e.g., Queen’s University) unaccompanied by evaluation research.

The authors of most reviews of rape prevention efforts conclude that theoretically-based programs targeted at single-sex audiences have more promise than mixed-sex programs [29,31,34,35]. The exception to this recommendation is seen in more recent educational efforts, where men and women in the audience are being approached as potential bystanders to situations that are developing rather than as perpetrators or victims [36-38]. Even in these circumstances, the same programming is normally offered to men and women in single-sex groups. Educating men to reduce their perpetration is a necessary goal but one that has so far been met with very limited success [39,40]. Until such time as there is widespread social change or better programming for men, effective rape resistance education (also known as risk reduction programs) for female students is the most promising option [41].

### Research on rape resistance education programs and guiding frameworks

Within the past fifteen years, a consensus view of the necessary elements of rape resistance education has emerged. Comprehensive critical reviews [29,35] stress the importance of the process of education and claim that effective rape prevention programs are likely to be those that actively involve participants and create a sense of high personal relevance. Building on social psychological theories of attitude change and persuasion, such as Eagly and Chaiken’s attitude change model [42,43], recommendations for program design include the use of expert status and positive consensus information to address cognitive, emotional, and behavioural aspects of attitude processes while ensuring personal motivation and decreased distractions and negative emotional states.

In 1996, Nurius and Norris [44] “offer[ed] a theoretical model that consolidates background, environmental, and intrapersonal variables related to women’s experience of sexual coercion in dating into a coherent ecological framework and present[ed] for the first time a cognitive analysis of the processes women use to formulate responses to sexual coercion” (p. 117). A key article synthesizing a decade of research on rape [41] based its recommendations for rape resistance education programs largely on the Nurius and Norris model expanding it with self-defence training based on research examining tactics predicting successful resistance, [45]. This program plan, named by them “AAA: Assess, Acknowledge, and Act”, expands on the cognitive appraisal model.

### Previous trials of rape resistance programs

A systematic review of the literature confirms that despite the size of the ‘at risk’ population and the seriousness of health impacts of rape and sexual assault, a rigorous randomized controlled trial (RCT) of a rape resistance education program has not been conducted. Among the published studies of programs for female university students, only three have demonstrated a positive impact on completed rape rates. However, they have mixed effectiveness and lack the detail to allow a full evaluation of the processes and practices [46,47].

In their study, Hanson and Gidycz [48] found lower completed rape rates for women in the subsequent two months, but the benefit was only for women who had not been sexually victimized previously. This benefit for non-victimized women was not replicated in a later study that attempted to strengthen the program [49]. Increasing the focus on cognitive and emotional obstacles to resistance also did not improve the outcomes [50]. In an expanded 3-hour program with an increased emphasis on personal relevance to participants and changes expected to enhance processing of information, Gidycz et al. [51] reported a delayed benefit (occurring between 2 and 6 months following the program) for some women only. Women who took the program, and who were sexually assaulted but not raped in the two months after the program, were less likely to be assaulted in the next 4 months than were any other group of women. Gidycz et al. [46] expanded their program to two sessions and added a booster session at 3 months. The 7-hour program included a greater focus on problem solving related to reducing risk and self-defence training but was unable to produce any change in sexual assault outcomes. Orchowski et al.’s [47] addition of self-defence training was successful in producing short term (2 month) reductions in completion of sexual assault for women with and without a sexual assault history. Effects were not maintained 4 months later even with a booster session. Yeater et al. [52] tested the efficacy of reading a “skills-based” self-help book against a wait-list control with 110 college women and demonstrated a number of positive outcomes but no reduction in sexual assaults.

The few other RCTs that also emerged were evaluations of treatment or prevention programs for sexual assault survivors. For example, an RCT of the effectiveness of a self-defence program for female veterans with PTSD following sexual trauma was conducted, but only a discussion of ethical concerns related to the project was found in the published literature [53].

### Development and pilot testing of a sexuality-enhanced AAA sexual assault resistance program

Based upon the models of Nurius and Norris [44] and Rozee and Koss [41], a three-session AAA sexual assault resistance program was developed by the principal investigator of the present trial and pilot tested in the fall of 2005 and winter of 2006 on a total of 34 female students against a quasi-experimental control [54]. Positive outcomes at 1 week for women in the intervention compared to controls included increases in the perception of risk, increased self-defence self-efficacy, and higher generation of effective self-defence strategies in hypothetical sexual assault and coercion situations; negative beliefs relating to rape myths and victim provocation in sexual assault were reduced. An unplanned 3-month follow-up with approximately 50% of participants revealed that 21% of women who took the program versus 39% of controls became victims of sexual coercion or assault. Based upon these results, a revised AAA program was conceived to include additional self-defence scenarios and practice.

Rape resistance programs alone may not be enough to protect women from sexual assault in the longer term. Normative sexual practices which put men’s (presumed) sexual needs ahead of women’s have also been implicated in acquaintance sexual assault [55]. For an education program to have long-term effectiveness, it is important to integrate what women know about rape and sexual coercion into the development of their healthy sexuality and relationship practices. The Our Whole Lives (OWL) Curriculum [56], was designed: “to advance communication about sexuality and help participants address their own needs … ; to promote safer sexual practices; to build understanding of healthy sexual relationships and activities; … [and] to help participants accept, understand, and affirm their own sexuality…(p. xi)” Because these principles lay the groundwork for a positive sexuality from which unwanted sex may be resisted more successfully, an adaptation of some of the OWL curriculum units was included in a sexuality-enhanced version of the AAA program.

In 2006–2007, a small RCT of the revised AAA program versus a sexuality-enhanced version of the AAA program versus a no-program control was conducted with 214 students [57]. Women who were assigned to the AAA programs had 40 to 50 percent lower rates of completed sexual assault at 3 and 6 months than women who were not (3 month: 12% control, 6% program; 6 month: 8% control, 5% program). When sexual assault history was included as a covariate for the secondary outcomes, the results did not change substantially, suggesting that the program was equally effective for women with and without a history of prior victimization. In addition to this positive outcome, integration of broader sexual education into rape resistance education had unique benefits related to greater perception of risk.

Notwithstanding these successes, the sexuality unit was presented first, followed by the three sexual assault resistance units and we found unacceptably high attrition between the sexuality session and the sexual assault resistance units (25-30%) compared to attrition between units when sexual assault resistance was offered alone (0-23%). Interviews with the women who dropped out of the combined program suggested that there were two main reasons for this elevated attrition rate. There was discomfort with the sexuality content in the absence of a real bond with the facilitators and the group, and disappointment because the first unit was not sexual assault resistance. Based upon these interviews and discussions with other researchers and educators, the sexuality-enhanced version of the AAA program was revised to offer the sexuality unit last and to contextualize it as an integration of the sexual assault resistance knowledge into women’s on-going and future sexual and romantic relationships. In a subsequent pilot study in 2007–2008 with 32 students, this revision resulted in comparable attendance (82%) for the sexuality unit as for any of the other AAA units.

### Aims

#### Primary

To test the hypothesis that a novel, four-session, small-group sexual assault resistance education program can reduce the one-year incidence of sexual assault by 30% (absolute difference of 7.5%) among first-year female university students, when compared to current university practice.

#### Secondary

To test the hypotheses that the intervention can: (a) increase women’s perception of their risk of sexual assault by male acquaintances; (b) increase women’s belief that they could defend themselves if confronted with a sexual assault situation; (c) increase women’s knowledge of the most effective self-defense strategies; (d) increase women’s ability to detect risk in hypothetical situations; (e) reduce women’s rape beliefs and attitudes that impair recovery from sexual assault.

#### Tertiary

To test the hypothesis that the intervention can: (a) reduce the one-year incidence of forced sexual contact and sexual coercion; (b) maintain the improvements in the primary and secondary aims for up to 24 months.

## Methods/Design

### Study design

This is a multicentre randomized controlled trial. By necessity, the design will be open-label, whereby the participants will be aware of the nature of the assigned sexual assault intervention. There is no evidence from our pilot testing that knowledge of the assignment influences participants’ responses on key outcomes in any way. For example, post-randomization pre-intervention scores did not differ between participants who were assigned to the AAA education programs and those assigned to no-program.

### Inclusion and exclusion criteria

Inclusion criteria: (a) female subjects; (b) age 17 to 24 years; (c) first-year university student (completed no more than 10 university courses); (d) able to attend one of four scheduled programs in the semester they are enrolled; (e) provide informed consent. There are no exclusion criteria. Universities require that all attending foreign students have English proficiency; therefore all students will have sufficient English to participate.

### Recruitment

Women will be recruited from each of three sites over a 2-year period, Universities of Windsor, Guelph, and Calgary. The universities are in large and small cities, attract different demographics of students and have very different first year cultures. For example, the University of Windsor is in one of the most ethnically diverse cities in Canada, at the University of Guelph almost all first-year students live on campus, and at the University of Calgary less than 10% of first-year students live on campus. We expect total recruitment from all three sites of more than 200 each fall and winter academic term from contacted students and 50 from orientation activities or first year classes. Our pilot testing indicated that the projected rates of recruitment are achievable. When women were contacted in our pilot study, no more than 11% of them declined and this was primarily due to time considerations rather than topic.

Although recruitment will be site-specific, the information provided to participants in advertisements and in contact with the recruiters will be standardized. Recruitment will begin each year in September with e-mail contact of first year female students by the Registrar, Faculty, or Director of Student Residence. The e-mail will consist of a brief description of the study and the incentives offered and will ask those interested to contact the research assistants by telephone or e-mail. Lists of eligible women in Psychology Participant Pools will also be obtained and research assistants will contact these women. Other advertising will be done by posters in female residence halls and in women’s washrooms on campus, on electronic and physical research posting boards, and flyers left in residence mailboxes. Also, face-to-face recruitment will take place in large first-year classes, at tables in student centres and at orientation and other events.

All interested students will be asked to contact a research assistant who would explain the study in detail prior to scheduling the baseline session. Women will be screened by telephone whenever possible. In instances where women cannot be contacted by telephone they will be provided a detailed description of the study and eligibility criteria by e-mail. All prospective participants will be contacted within the early part of the fall and winter terms. Those who agree to participate will be invited to attend an in-person baseline assessment matching their chosen intervention schedule.

### Randomization

The trial biostatistician will set up the centralized randomization using an SSL secured, password-protected web-based technology. Site will be the only stratification factor for administrative reasons. At the time of the recruitment dialogue, the research assistants will be unaware of the arm to which a participant would be assigned. Following signed consent, verbal confirmation of eligibility, and completion of baseline measures, the research assistant will log onto the randomization website, enter each participant’s code number, and will receive the random group assignment. Based upon the assignment, the research assistant will direct the participant to the room where their first intervention session will take place without revealing their assignment to them. Great care will be taken to ensure that agreement to participate and selection of the intervention schedule will be obtained prior to random assignment so that participation could not be affected by the recruiter’s or participant’s knowledge of assignment.

### Interventions

#### Control condition: University-provided brochures

To match the current ‘standard of care’ at Canadian universities, participants will be invited to take and read brochures on sexual assault selected from those available on their Canadian university campus. It is not possible to provide identical brochures across all three sites as each contains city-specific community services and sexual assault hotlines. However, the content of the brochures regarding sexual assault are similar and all sites’ brochures include general information on sexual assault and ‘date-rape’ drugs and post-rape legal and medical advice. One of two research assistants at each site will conduct each session, asking the participants to take brochures they are interested in, read them over, and ask any questions they may have. Questions will be answered in a group setting or individually if a participant stays after other participants have left to ask their question privately. Interaction between participants and the research assistant on the topic will be limited to 10–15 minutes after all preliminary activities (e.g., settling, getting food or beverages) are completed and will be audio recorded for verification.

#### Intervention condition: Enhanced AAA Sexual Assault Resistance program

The program consists of four separate units which use a combination of information-providing games, mini-lectures, facilitated discussion, brainstorming, large and small group activities, application and practice with DVD and audio clips, written scenarios, and role plays [57]. Participants will have a choice of either 1 unit per week over 4 weeks or all 4 units in one weekend (2 each day). At the first session, which will immediately follow the baseline session and randomization, participants will receive a resource kit including brochures and other resources. All sessions will be audio recorded.

##### **Unit 1**(3 hours)

The ‘Assess’ component focuses on improving assessment of risk for sexual assault in situations involving male acquaintances and developing problem-solving strategies to reduce risk. Personal relevance is heightened through the use of local statistics and a physical demonstration of the probability of sexual assault for women in the room. Sexual assault definitions and laws are presented briefly. ‘Assess’ is also related to the part of the process where the woman has communicated that she does not wish to engage in a sexual act and the man ignores her wishes and “continue[s] to touch, bully, or threaten” [41], p. 299. Beginning in Assess and developed further in later units, women are trained to identify this situation as dangerous and to take into account aspects of the situation such as isolation, possible escape routes, etc. Considerable time is spent providing women with empirically-based information on environmental/situational cues (e.g., alcohol) and men’s behaviour danger/risk cues [58], connecting these to gender and dating stereotypes, and giving women practice identifying increased risk and coming up with non-restrictive ways around it (i.e., ways to reduce the potential perpetrator’s advantage while still enjoying social situations).

##### **Unit 2**(3 hours)

The ‘Acknowledge’ component assists women to recognize more quickly the inherent danger in situations that have turned coercive and to explore ways to overcome emotional barriers which can prevent women from engaging in forceful resistance against known men. “Reluctance to label the situation as rape slows her protective response” [41], p. 299 and most women who are assaulted do not label their experience as rape [59]. In this unit, women’s personal sexual rights are reinforced. Exercises facilitate the exploration of the sometimes competing goals in social situations such as the desire for romantic (or friend) relationships and the need for personal safety [60]. The facilitators debunk the miscommunication hypothesis i.e., the argument that women are not clear enough in their sexual refusals or do not mean “no” when they say “no;” [61,62], and provide practice for women to emotionally prepare for and respond to common verbal coercion strategies [63].

##### **Unit 3**(3 hours)

The ‘Act’ component presents a range of potential options for resistance depending on elements of the situation, the man’s actions, and the success of early strategies. Typical self-defence strategies are often rejected by women because they are reluctant to use tactics such as “keys in the eye” against a man they have been dating or know socially. In fact, the strategies that women are most likely to use in acquaintance rape situations are precisely those that men say they are most likely to ignore [64] and which are least effective [45,65,66]. There is a brief discussion of the myths that undermine women’s resistance (e.g., the unsupported claim that women who defend themselves are hurt worse) [67]. Research evidence on the ineffectiveness of certain tactics and the effectiveness of forceful verbal and physical resistance are provided [45,68-70]. Each woman is encouraged to create a ‘tool box’ of forceful strategies that she would be willing to employ against a man she knows who is threatening her sexual integrity, and to escalate her resistance if any early strategies she tries are ineffective. Physical self-defence training (based on WenDo) includes standard instruction in making a fist, yelling in a way that fuels self-defence efforts, assessing vulnerable parts of the body, as well as techniques for punching and kicking. Additional strategies focus on defending against men in more common acquaintance situations (e.g., breaking wrist or choke holds, getting out from under someone using body weight to restrain you). This component also includes discussion of the need to overcome the emotional barriers to forceful physical defence against male acquaintances when the threat demands it.

##### **Unit 4**(3 hours)

This unit adapts content from *The Our Whole Lives* (OWL) sexuality education curriculum [56] and takes what women have learned from the previous three units and applies them in a more focused way to longer term romantic and sexual relationships. The unit is designed to assist participants to become more comfortable talking about sexuality, to expand discussions of sexual practices beyond intercourse, facilitate identification of women’s own sexual values and desires, sexual boundaries, and safety needs, and provide practice in communicating this knowledge in an assertive and self-efficacious manner, as well as increase women’s overall understanding of what healthy sexual relationships mean to them. This ensures that the education program is more fully integrated into participants’ lives.

### Assessments

At baseline, participants will complete computerized surveys prior to being randomized. They will then go immediately to their first intervention session, either to receive their brochures or to attend their first educational unit. To assess knowledge acquisition, intervention participants will have their 1st follow-up assessment 1 week after the last intervention session with control participants matched to the same interval. Subsequent follow-up assessments will occur at 6, 12, 18, and 24 months from the date of randomization. We will measure outcomes using in-person computerized surveys at baseline and 1 week after the last intervention session. All other follow-ups will use secure web-based surveys linked only by participant codes to protect privacy.

### Outcome measures

#### Primary

Completed sexual assault (rape) will have occurred when a participant indicates she has had at least one experience of sexual intercourse (oral, anal, or vaginal) that was threatened, forced, or drugged (completed, not attempted) in the period between the previous outcome measurement and the present; answered ‘once’ or more to any of 9 questions (involving oral sex, vaginal penetration, and/or anal penetration “without consent” when the woman was “too drunk or out of it to stop what was happening”, was threatened with physical harm to herself or others she cares about, and/or was forced, “for example holding me down with their body weight, pinning my arms, or having a weapon”) on the *Sexual Experiences Survey* - *Short Form Victimization* SES-SFV, [71]. Month of occurrence will be recorded to permit time-to-event analyses. Previous versions of the SES have been used in large university, college, and community samples, and it is considered the gold standard. Responses on the scale are stable across administrations. Test-retest reliability is very high, and responses are also comparable to those received through interviewing women [72]. Concurrent validity has been demonstrated using correlations between sexual victimization as measured with the SES and variables predicted to be affected or influenced by that victimization, for example, hostility toward men [73,74]. Construct validity has been demonstrated when women’s descriptions of sexual experiences were rated by interviewers (on the items of the SES) and compared to the women’s own responses on the SES [75].

#### Secondary

The expanded cognitive appraisal model upon which the educational program is built outlines the process through which changes to the primary outcome are accomplished. Outcomes corresponding to secondary hypotheses (a) through (d) measure these intermediary processes. The outcome corresponding to the secondary hypothesis (e) measures attitudes likely to be affected by the program and is related to successful coping with sexual assaults. These will allow targeted revision to the program if necessary.

(a) Perception of risk of sexual assault by male acquaintances will be measured by a single item, “What are your chances of being raped by someone you know?” which has been adapted from Gray, et al. [76].

(b) Belief that women can defend themselves if a sexual assault situation were to arise (*Self-defense self-efficacy*: Marx et al.’s [77] adaptation of Ozer and Bandura [78] – higher scores indicate greater self-efficacy). This is a 7-item instrument with responses ranging from “not at all confident” to “very confident” on a 7-point scale. The instrument has a 0.83 Cronbach’s alpha level of internal consistency. Two items were also added to specifically measure a participant’s sense of her own ability to defend herself against unwanted sexual experiences, “How successful do you believe you would be in fighting off or otherwise stopping an attempted rape by a stranger?” and “How successful do you believe you would be in fighting off or otherwise stopping an attempted rape by a man you know (e.g., a man you are dating)?” Participants answer each of these two questions on a 7-point scale, ranging from *completely successful* to *completely unsuccessful*. These questions were pilot tested and found to have a 0.87 Cronbach’s alpha level of internal consistency.

(c) Knowledge of effective rape resistance strategies will be measured by identification of a greater number of forceful physical and forceful verbal strategies in response to threatening hypothetical situations (*Resistance measure*, [79]) and real situations (items requesting details following sexual assaults experienced, questions added to SES items, [48]). Testa et al.’s [79] measure follows a scenario depicting an interaction where the participant is on a date with “an attractive man” (p. 666). These items about the participant’s “intended behaviour” break down into 3 subscales: direct-resistance, polite-resistance, and passive-response. The direct-resistance subscale, which is our focus, has high internal consistency ranging from 0.89 to 0.94 and validity demonstrated by the authors. Additional, added questions, “If a man I knew (e.g., a date) tried to force me to have sex with him when I didn’t want to, I would …”, If a stranger tried to force me to have sex with him when I didn’t want to, I would …” were pilot tested to ensure clarity and consistency of coding of responses. Scoring is based on Ullman’s [45] study of the tactics used by women who avoided completed rape and has high inter-rater reliability (Cohen’s kappa > 0.90).

(d) Ability to realistically assess risk of harm in hypothetical scenarios where acquaintance sexual assault is likely will be measured using the: (1) *Risk perception* questionnaire [79]. Using the same scenario with “an attractive man” as the strategies measure above, the risk perception measure presents statements of 10 possible outcomes, 4 positive and 6 negative. The participants answer on a 7-point scale, from *not at all* to *very likely*, what they perceive the outcome of the interaction will be. Internal consistency is in excess of 0.85 by Cronbach’s alpha; (2) *Risk Perception Survey* (RPS) [80]. Two vignettes, one based on a stranger and one on an acquaintance situation “were designed to incorporate risk factors identified in the literature and included … clear risk factors and ambiguous risk factors” (p. 162). Participants are asked to identify the line number at which they feel uncomfortable (identify risk) and the point at which they would leave (take action). Higher scores indicate toleration of higher risk in the situation. These are the only measures available and once completed cannot be used again due to familiarity with the scenarios. As such, this outcome can only be measured 3 times (Testa’s once; Messman-Moore’s twice, once each for the stranger and acquaintance versions). The timing of measuring this outcome is spread out across the first year of follow-up.

(e) Beliefs and attitudes about rape will be measured by endorsement of rape myths (*Illinois Rape Myth Acceptance Scale* IRMA-SF,[81], and misinformation about the causes of rape (*Perceived Causes of Rape Scale* PCOR,[82,83]. The IRMA has the best psychometric properties of any rape myth scale, having excellent criterion-related validity (correlations with measures of sexism and hostility toward women) and reliability. Internal consistency is high (Cronbach’s alpha > 0.82). The PCOR “provides [an] assessment of a range of people’s beliefs about the causes of rape that are not exclusively rape myths” [83], p. 228. This 32-item instrument provides participants with the stem “Rape is caused by ....” and they answer how much they agree with a list of causes. Our focus is on the *Female Precipitation* subscale which has the best internal consistency. Test-retest reliability of 0.87 is also good.

#### Tertiary

Using the SES-SFV, forced sexual contact will have occurred when a participant answers one of three questions indicating she had experienced threatened, forced, or drug facilitated non-consensual sexual touch not including intercourse (e.g., “fondled, kissed, or rubbed up against the private areas of my body (lips, breast/chest, crotch or butt) or removed some of my clothes”). Sexual coercion will have occurred when a participant reports one or more incidents of verbally coerced (e.g., “threatening to spread rumors about me”, “continually verbally pressuring me after I said I didn’t want to” -- excluding threats of physical harm) non-consensual oral, vaginal, or anal intercourse.

### Proposed methods for protecting against sources of bias

#### Selection bias

Selection biases are evident in all studies and programs with a sexual or sexual violence theme because they disproportionately attract more sexually experienced and sexually victimized participants [84]. Similar selection biases are in effect when voluntary rape programming is offered on campuses. The internal validity of our intervention is high, and therefore selection biases are not likely to have a strong influence on the outcomes. Selection bias will be assessed by comparing the sample to the entering student demographic data.

#### Outcome assessment bias

Blinding is not possible in this trial. However, bias in outcome assessment is addressed by having the primary outcome, sexual assault, collected with the best measure of coercive sexual experiences available [SES-SFV, 71]. This measure will be applied equally to all study participants. Previous versions of the measure have been tested on tens of thousands of undergraduate students and it has high reliability and validity [72,85]. The 2007 scale corrects weaknesses in the earlier version. Although the SES-SFV is not an ‘objective measure’ of sexual assault, since no external adjudication is possible, it is the best option available. The instrument does not ask about the occurrence of sexual assault or coercion directly but rather asks how often a particular behaviour occurred (e.g., “A man put his penis into my vagina, or inserted fingers or objects without my consent by using force, for example holding me down with his body weight, pinning my arms, or having a weapon”).

#### Differential drop out

Our pilot study indicated that dropouts are minimized by having follow-up times coincide with the school year rather than with holiday breaks. Based on Dillman [86] we will make up to 4 reminder phone calls/e-mails following confirmation of contact at follow-up. Further, web-based survey administration makes completion of surveys easier for participants, as measured by our low drop-out rate (3 mo, 0%; 6 mo 11%) in the 2007/2008 pilot study. A research assistant at each site will be hired to phone and e-mail participants to complete follow-ups. For students who do not wish to continue in the study, we will administer a 5-minute telephone version of the SES-SFV at 1-year and 2-years so we can ascertain their sexual assault status. Taken together, these methods will reduce the drop-out rate considerably.

There is a possibility that women in the control arm may not feel as connected to the trial, and may drop out as a result. Our pilot data are suggestive of this possibility. We have reduced the likelihood of differential drop out by providing a small intervention (brochure) rather than using a no-contact control, by providing incentives and personalized phone contact, as well as offering the opportunity for control participants to receive the full program at the end of the trial. These maneuvers will maximize connection to the trial and will reduce differential attrition.

#### Lost to follow up

Lost to follow-up will be defined as not being able to ascertain a woman’s sexual assault status at 1 year. Our 6-month pilot study had less than 5% of participants moving or changing their phone numbers or e-mails without updating their information with the Registrar or contacting the researchers, and therefore being ‘lost to follow up’. The primary outcome for the present trial is at 1 year rather than at 6 month follow-up, and therefore we have doubled the estimated rate of lost-to-follow-up to 10%. We expect a loss of no more than another 15% at the end of the second year of follow-up. Our lost-to-follow-up numbers are reasonable based on recent estimates that less than 25% of students drop out of university by the end of their second year [87].

#### Compliance

Non-compliance (i.e., “no show”) will be defined as not receiving any brochures or not attending the education program. From our previous pilot studies, as many as 40-50% of women who completed baseline measures did not show up to receive their intervention which was scheduled at a later date. With a time-gap between baseline assessment and intervention, we were not able to reduce no-show below 40% with any techniques we tried in pilot tests. Therefore, for the present trial we will be pre-testing and randomizing on the same day, followed immediately by the (first or only) intervention session (and a meal) which should reduce non-compliance to near zero. In order to minimize attrition once the education intervention begins, enrollment into the trial will start close to the beginning of the term or immediately following the midterm period. We will also use incentives and reminder phone calls. The incentives for program/control session attendance include a ballot for a final study-end lottery of $300, a $25 movie pass, and a free lunch/dinner. To retain participants in the remaining education program units, reminder calls will be used, as well as, bonus points, refreshments, a cash lottery for each session ($50), a ballot for the study-end lottery for every session attended, a colorful clipboard filled with resources, a themed magnetic note board, and a completion certificate for full compliance. Using a lower level of incentives in our pilot study, we were able to attain full attendance at the educational program from more than 78% of participants.

Non-response on follow-up surveys will be defined as refusing to complete portions of or all secondary outcome follow-up surveys. We will use a number of procedures known to maximize response rates for follow-up surveys [86] such as confirmation of contact information at follow-up and multiple reminder calls, emails, and/or letters. Additionally, participants will be asked to provide long-term and collateral contact information at the post-intervention survey and at each subsequent follow-up survey. Specifically, they will be asked to provide primary and secondary email addresses, primary and secondary telephone numbers and their addresses during the summer (where applicable), and their future address if they are planning to move in the upcoming year. Participants also will be asked to provide the name, telephone number, and email address of at least two people who would be likely to know their whereabouts in the event that their contact information changes.

Our pilot studies also made it clear that attractive incentives must be in place so that participants stay in the trial for its full duration after the intervention is complete. We have chosen $30 gift certificates for survey completion based upon other studies in the literature [46,80] and our own pilots. We will also, based on participant feedback, be using web based surveys (with all appropriate security and privacy measures taken) for the follow-ups for this trial, which we found improved the ease with which women could participate and resulted in 100% 1 week and 89% 6 month completion ($20 incentive) for the 2007 pilot study. Moreover, the follow-up outcome surveys will take less than 30 minutes to complete which will maximize response rates [86].

The use of incentives is standard in psychological research and, increasingly, in clinical trials as long as the size of the payment cannot be seen to put ‘undue’ pressure on individuals to participate, and the sample is not a ‘vulnerable population’ e.g., [88-90]. Our pilot testing prior to the first program offering has confirmed that our lotteries (which keep down costs) are perceived positively by potential participants but that the size of the lottery ($500 total) does not work to induce an otherwise non-interested or unwilling person to consider participation. As such the $500 in lotteries is a useful incentive to strengthen existing interest without coercion. Our $25 movie passes for early survey sessions and $30 gift certificates for the follow-ups are well within the range clearly considered modest (<$100) and acceptable by authors publishing in *Contemporary Clinical Trial*[88]. With no incentives within a student population, our ability to recruit and retain participants in the longitudinal research would be extremely limited.

While cross-contamination effects are not likely to affect this kind of intervention [91], we will ask the women in the control arm at the 1-year follow-up the following questions: “Have you had conversations about sexual assault with other women?” “If yes, was this woman in the other longer program?” “How long was your conversation(s)?” “What did you talk about?” We will describe the amount of ‘contamination’ occurring and discuss its possible influence on the findings.

#### Training of facilitators and research assistants conducting interventions

A detailed manual has been written that provides instructions for the enhanced AAA sexual assault resistance program facilitators, including session materials, scripts, and trouble-shooting advice. The Principal Investigator will conduct a joint training program for all site facilitators in late summer in the intervention years of the trial. Training will include attendance at a WenDo Women’s Self-defense two-day Basic program and another day of specialized training by a certified WenDo instructor. Research Assistants will be trained by the Principal Investigator and the Trial Project Manager in site visits in September in both intervention years.

### Sample size considerations

The primary outcome is completed sexual assault (rape). Our 2006–2007 pilot randomized controlled trial of 214 students yielded the following cumulative incidences. For women in the control arm, the 3- and 6-month rates were 12% and 20%, respectively. For those in the sexual assault resistance education program, they were 6% and 11%, respectively. These figures correspond to a 50% relative risk reduction. This is comparable to the one previous study conducted by Hanson and Gidycz [49], which yielded a rate of 14% of rapes in their control arm and 6% in their intervention arm over a 3-month period, among women with no history of previous victimization. Given that the incremental incidence of sexual assault declines over time we estimate, for the purpose of sample size calculations, the 1-year rates to be 25% in the brochure arm and 17.5% in the educational program arm; a more conservative 30% relative risk reduction. Therefore, a sample size of 932 women (466 per group) will have 80% power to detect an absolute difference of 7.5% (25% versus 17.5% equals a 30% relative reduction) at a two-sided 5% level of significance. This is a meaningful reduction because it implies that for every 14 women who enroll in the program, 1 additional rape could be averted. Aside from power considerations, it is important to note that a sample of this size will yield a significantly positive trial when the observed difference between groups is as small as 5.4% (21.6% relative risk reduction). Although the intracluster correlation was calculated as −0.02 from our pilot study, +0.02 will be used to inflate the sample size with a design effect of 1.38 and a cluster size of 20 women. Assuming a lost-to-follow-up of 10% in the first year and 15% in the second year of follow-up, the total size of the trial will be 1716 women (932×1.38÷0.75).

### Statistical analyses

Analyses will be performed after all women have completed their one-year follow-up and at the end of the trial. Following the intention-to-treat principle, a participant will be included in the analysis if she is randomized. Using SAS software [92] the primary (and tertiary) analyses will compare the proportion of women with completed sexual assaults between groups using a Wald test from a log-binomial regression model (PROC GENMOD) that adjusts for compound symmetry (CS) clustering using generalized estimating equations. Covariates will be added to the model if differences among baseline characteristics are observed. In addition, a test for interaction will be performed using the above model to assess whether there is a differential treatment effect depending upon whether the women participated in the weekend versus weekday programs. Women who are lost to follow-up or drop out can affect the validity of the group comparison if (a) sexual assault is related to being lost or dropped and (b) it is differential between groups. Thus, baseline characteristics of women who are lost and drop out will be compared to women who complete the trial. In addition, the missing data mechanism will be formally tested [93] to determine whether it is Missing Completely at Random (MCAR) or is Missing at Random (MAR). Freedom from completed sexual assault will be compared between groups using Cox regression (PROC PHREG) with a robust sandwich covariance matrix to account for the clustering. Women who are lost to follow-up or drop out will have their data censored at the time of last contact.

For the secondary outcomes which are all continuous (or at least ordinal) longitudinal, repeated measures analysis of covariance (ANCOVA) will be used to compare intervention arms across the follow-up periods, adjusting for baseline scores on the instruments. Generalized linear models (PROC MIXED) will be used to correctly adjust the variance estimates for CS clustering. Recognizing that some of the instruments have ordinal responses, a rank-based ANCOVA will also be performed to assess congruency with the parametric analyses. For completeness in assessing the effect of missingness, two additional analyses will be conducted: (a) a ‘per-protocol’ analysis among participants who attend all sessions and respond to the 1-year SES-SFV questions; (b) multiple imputation of five data sets (PROC MI and MIANALYZE) using a discriminant function for binary outcomes and Markov chain Monte Carlo for continuous outcomes, assuming the missing data mechanism is MAR. The literature, for example [49], suggests that prior sexual assault can be a modifier with respect to the effectiveness of sexual assault resistance programs. Accordingly, program effects will be compared for women with and without sexual assault histories at baseline.

### Ethical considerations

Ethical approval for the trial was received from the University of Windsor Research Ethics Board on May 31, 2011, from the University of Guelph’s Research Ethics Board on June 14, 2011 and from the University of Calgary’s Conjoint Health Research Ethics Board on September 9, 2011. There are no serious anticipated risks to participants, although the content of the educational interventions and some survey questions on sexual assault could bring up negative feelings for participants, particularly if they have an assault history. The self-defence instruction adds limited physical risk if participants do not follow instructions carefully. In our pilot studies involving over 200 women, none reported adverse effects.

### Steering committee and trial oversight

The Steering Committee for the trial will consist of the Principal Investigator and four Co-Investigators. A Data Safety Monitoring Committee is not needed as our psychosocial intervention does not warrant it. The Trial Project Manager will make decisions for all sites on trial issues that arise and communicate with the Site Coordinators to ensure consistency on these issues. The Trial Project Manager will be supervised by the Principal Investigator and the Site Coordinators will be supervised by the Site Investigators. Research Assistants will be supervised by Site Coordinators. The Trial Project Manager, Principal Investigator, and Co-Investigators will meet monthly by teleconference and annually in person.

Intervention conformity will be measured by having site facilitators complete a Session Protocol Issues form at the end of each unit and by review of audio recordings of randomly selected sessions for each facilitator. Audio recordings will be reviewed by the Trial Project Manager and any outstanding issues will be addressed in special meetings. Protocol issues identified by facilitators will be addressed in weekly meetings.

### Trial status

The first participant was randomized on September 28, 2011. Currently, 918 women have been enrolled into the on-going trial.

## Discussion

On university campuses, sexual assault rates are high, especially among first- year female students. Therefore, these young women represent a critical population for resistance education interventions. There is now a consensus that relying solely on coeducational or men’s programs is insufficient to fully protect women from sexual assault. Programming that empowers women with knowledge, skills, and practice to resist coercive sexual behaviours from known men is needed. The proposed randomized controlled trial is an evaluation of a sexual assault resistance education program, designed to reduce sexual assault among young women in the first year of university. We believe that reductions in sexual assault will have a direct impact on the mental and physical health of all women at university, but particularly for those who are able to successfully resist completed sexual assault.

The results of the trial will provide a unique contribution to the published literature, in addition to producing a maximally effective sexual assault resistance education program and an accompanying facilitator training manual which can be adopted by universities. The trial results will also provide direction for further research into which aspects of the program need to be strengthened, as well as how long the effects of the program last and will indicate at which point in time refresher sessions may be necessary. If the program is effective, this may also provide an entry to implementation and research in high school and to other populations of young women who are not enrolled in university.

## Competing interests

The authors declare that they have no competing interests.

## Authors’ contributions

CYS, ME, PCB, WET, and IRN contributed to the overall design of the trial and participated in the acquisition of funding. All authors are involved in its ongoing management. KLH is responsible for the logistical aspects of the trial. All authors read and approved the final version of the manuscript.

## Pre-publication history

The pre-publication history for this paper can be accessed here:

http://www.biomedcentral.com/1472-6874/13/25/prepub
